# Supplementary material for: DciA is an ancestral replicative helicase operator essential for bacterial replication initiation
Source: Nat Commun. 2016 Nov 10;7:13271. doi: 10.1038/ncomms13271 (PMC5109545; doi:10.1038/ncomms13271)
Supplement: Supplementary Information — Supplementary Figures 1-4 [file ncomms13271-s1.pdf]

a

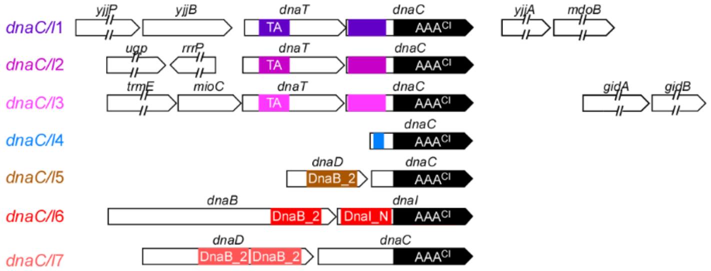

b

accessory protein	NTD size (aa)	NTD motif/domain	DE	domestication order
DnaC/I1<br>DnaC/I2<br>DnaC/I3	~ 70	-	3	Enterobacteriales<br>Alteromonadales<br>Aeromonadales
DnaC/I4	< 20	-	1	Aquificales
DnaC/I5	< 20	-	1	Chloroflexi
DnaC/I6	~ 90	DnaI_N	1	Bacilli/Tenericutes
DnaC/I7	~ 150	-	1	Clostridia

Supplementary Fig. 1. Genomic context of the seven domesticated *dnaC/I* identified. a. The domain AAA^{CI} common to all *dnaC/I* genes is shaded in black. The color code indicates the features that distinguish the seven genomic *dnaC/I* genes: the genomic context, the N-terminal domain (NTD) of *dnaC/I* and the companion genes. Pfam motifs identified; TA: toxin/antitoxin Phd_YefM motif, DnaB_2 : Replication initiation and membrane attachment, DnaI_N: primosomal protein DnaI N-terminus. **b.** Types of DnaC/I proteins. Characteristics of the N-terminal domain of DnaC/I proteins (NTD) are indicated. When available, the Pfam A domain is given. DE: Domestication Events.

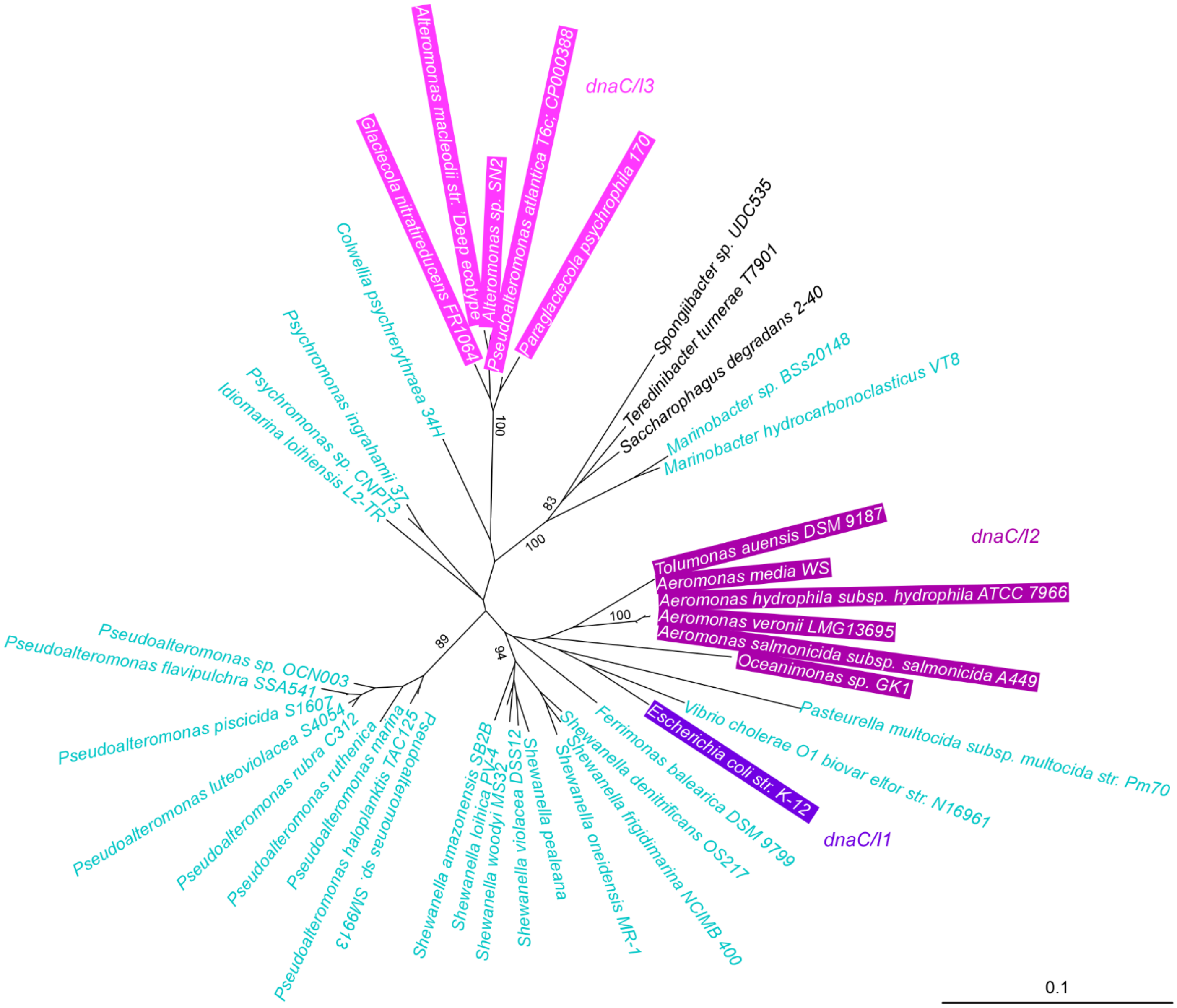

Supplementary Fig. 2. *dnaC* was domesticated thrice in the gammaproteobacteria. 16S-based phylogenetic tree of Alteromonadales and Aeromonadales (Methods). Only genomes, whose sequence is complete, were considered. *E. coli*, *Vibrio cholerae* and *Pasteurella multocida* were included as outgroup organisms. The organisms in which a copy of *dnaC* was domesticated are boxed in lilac (*dnaC/I1*), dark pink (*dnaC/I2*) and light pink (*dnaC/I3*). *dciA*-containing organisms are in cyan. Three organisms neither contain *dciA* nor a domesticated copy of *dnaC* (black); they were recently reclassified as Cellvibrionales [14]. Significant bootstrap values are indicated. Scale bar represents 0.1 substitution per site.

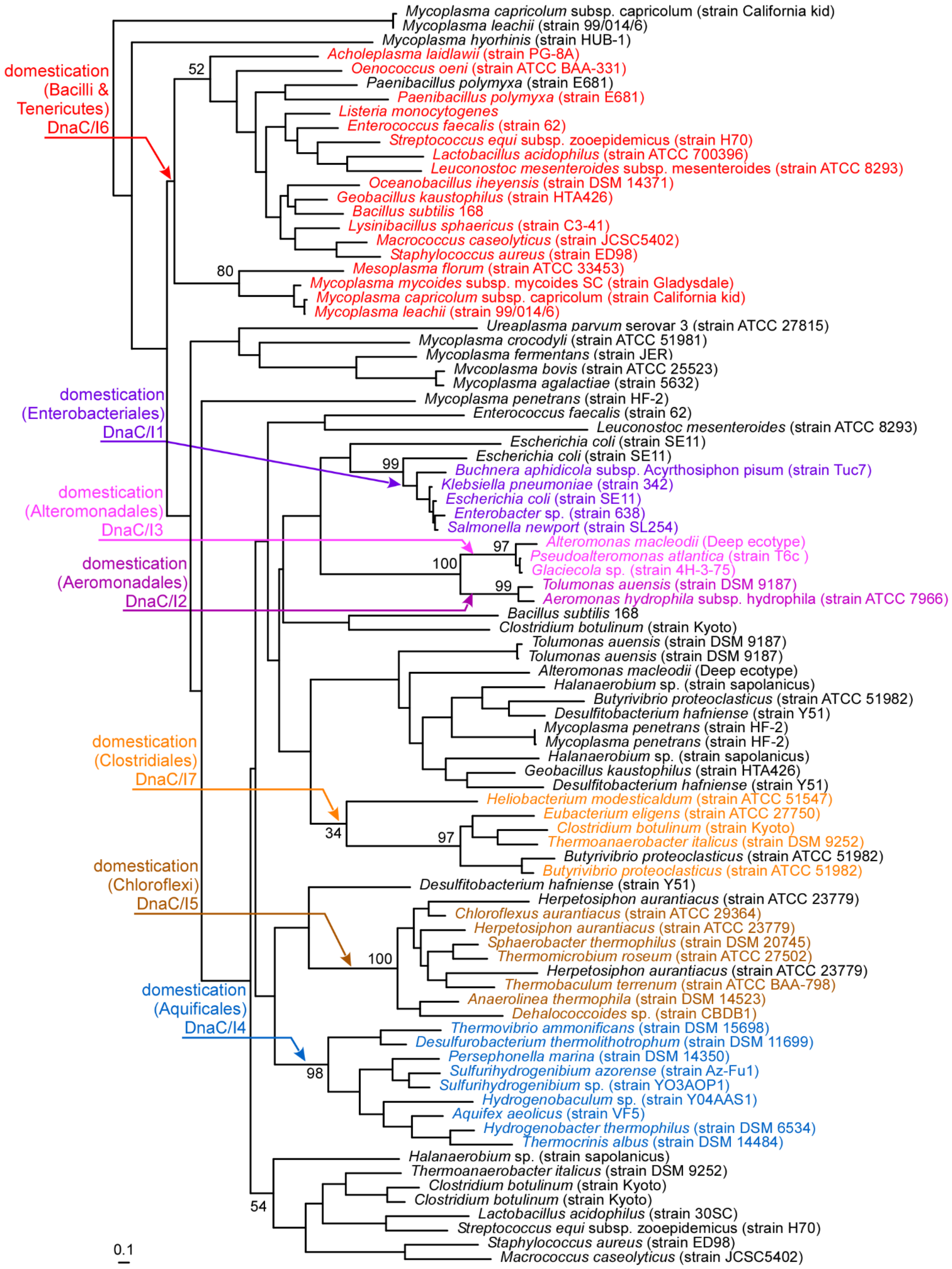

Supplementary Fig. 3. Seven domestication of *dnaC/I* within the bacterial domain. The sequence of representatives of each of the domesticated DnaC/I proteins (1 to 7) and of a few proteins expressed from mobile AAA^{CI}-carrying genes were collected to construct this phylogenetic tree. ϕ: phage, τ: transposon, gi: genomic island, π: plasmid. Bootstrap values of interest are indicated. Scale bar represents 0.1 substitution per site.

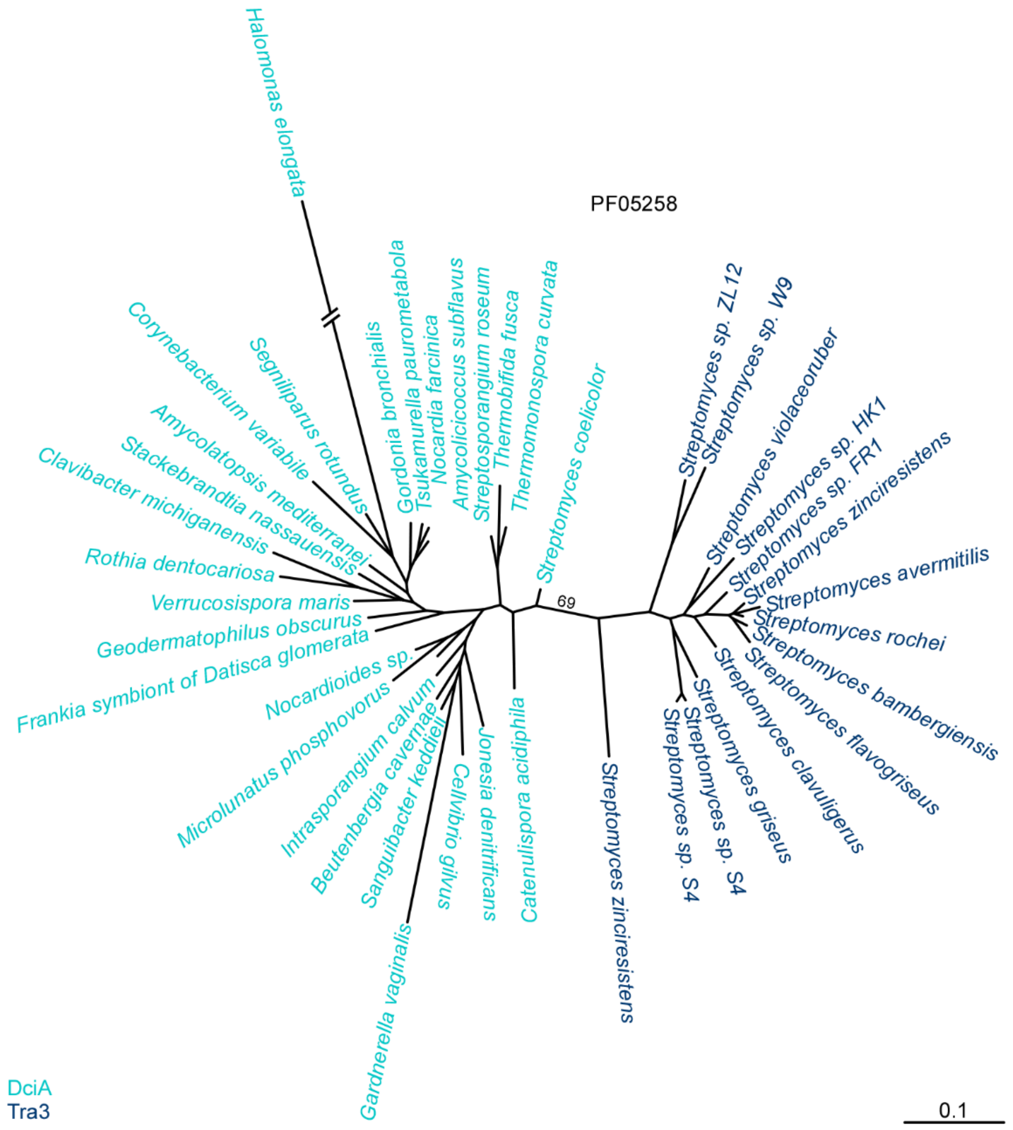

Supplementary Fig. 4. *dciA*-related gene are associated with plasmids in the genus *Streptomyces*. A phylogenetic tree, based on the various sequences of PF05258-carrying proteins of *Streptomyces* (the genomic *dciA* of *Streptomyces coelicolor* is presented as a representative of the genus *Streptomyces*) and species representative of Actinobacteria, was constructed (Methods). Three features distinguish genomic *dciA* genes (organisms in cyan) from the *tra3* genes (organisms in blue). Genomic *dciA* are located between *recF* and *gyrB* within Actinobacteria, whereas the localization of *tra3* is variable and always on a plasmid. *Streptomyces* species that contain a *tra3*-carrying plasmid contain also a genomic *dciA*. All *tra3* found in *Streptomyces* derive from a common ancestor and branch length between the different Tra3 in the tree reveals that relatively little pressure of selection has been applied on the sequence of the gene. Altogether, *tra3* may be considered a “mobile” PF05258-carrying gene. *Halomonas elongata*, which is a gammaproteobacteria, is used as an outgroup species. The bootstrap value associated with the branch from which emerges the group of mobile *dciA* is indicated. Scale bar represents 0.1 substitution per site.
